# Content validity of measures in early numeracy in children up to eight years: A COSMIN systematic review

**DOI:** 10.1371/journal.pone.0308874

**Published:** 2024-09-19

**Authors:** Renée Speyer, Airi Hakkarainen, Sangwon Yoon, Jae-Hyun Kim, Catriona Windsor, Sarah Wilkes Gillan, David Littlefair, Reinie Cordier

**Affiliations:** 1 School of Health Sciences, College of Medicine, Nursing & Health Sciences, University of Galway, Galway, Ireland; 2 Curtin School of Allied Health, Faculty of Health Sciences, Curtin University, Perth, Western Australia, Australia; 3 Department of Special Needs Education, University of Oslo, Oslo, Norway; 4 Department of Education, University of Jyväskylä, Jyväskylä, Finland; 5 Department of Education, University of Helsinki, Helsinki, Finland; 6 Department of Linguistics, Macquarie University, Sydney, New South Wales, Australia; 7 Discipline of Occupational Therapy, Faculty of Medicine and Health, The University of Sydney, Sydney, New South Wales, Australia; 8 Department of Social Work, Education and Community Wellbeing, Faculty of Health and Life Sciences, Northumbria University, Newcastle upon Tyne, Tyne and Wear, United Kingdom; 9 Department of Health & Rehabilitation Sciences, Faculty of Health Sciences, University of Cape Town, Cape Town, South Africa; UFSCar: Universidade Federal de Sao Carlos, BRAZIL

## Abstract

Early numeracy skills are considered essential predictors for later mathematical and educational achievement. Therefore, there is a need for early numeracy measures with psychometrically sound properties. This systematic review aimed to determine the content validity of all current early numeracy measures in accordance with the COnsensus-based Standards for the selection of health Measurement INstruments (COSMIN) framework and methodological guidelines, and was conducted and reported by following the Preferred Reporting Items for Systematic Reviews and Meta-Analyses (PRISMA) 2020 statement and checklist. Systematic literature searches were conducted in January 2024 in five electronic databases: CINAHL, Embase, Eric, PsycINFO, and PubMed. Eligible measures assessed numeracy, targeted children up to eight years of age, were published in English in 1995 or later, and had psychometric data on measure dimensionality. Eligible psychometric reports that were published in English described instrument development and/or content validity of included measures. The measures’ methodological quality was assessed using the COSMIN Risk of Bias checklist, after which all three aspects of content validity (i.e., relevance, comprehensiveness and comprehensibility) were evaluated. Six early numeracy measures and eleven psychometric reports were included. None of the measures could be recommended for use in clinical practice, education, or research due to a lack of high-quality evidence on content validity. However, no high-quality evidence was found to indicate insufficient content validity, therefore, all measures still have the potential to be used. Limited access to measures in the domain of early numeracy, despite having contacted both publishers and instrument developers, may have negatively impacted the completeness of the current overview of content validity of early numeracy measures. In line with the COSMIN guidelines, after the initial evaluation of content validity, future studies should evaluate the remaining psychometric properties of the included measures to identify the most robust measures in terms of validity, reliability, and responsiveness.

## Introduction

Early numeracy is an umbrella term for a multidimensional phenomenon [1] that encompasses several skills, including verbal counting, knowing the number symbols, recognising quantities, discerning number patterns, comparing numerical magnitudes, and manipulating quantities [2] Basic numerical competencies are considered essential predictors for later mathematical and educational achievement [3, 4]. Many models of early numeracy have been published, indicating a broad consensus in considering skills such as counting, number relations, and basic arithmetic as central aspects of early numeracy [5]. One of these models was specifically developed to support educators in identifying and supporting children at risk of developing mathematical learning difficulties. The four-factor working model of core numerical skills comprises symbolic and nonsymbolic number sense, understanding mathematical relations, counting skills, and basic arithmetic knowledge [6]. Still, further research is required to reconcile the differences between existing models, and to better establish how counting and ordering skills should be considered within them [5].

Educators must have access to psychometrically robust assessments to identify children at risk of early numeracy difficulties. A recent review on the quality of outcome measures used in early numeracy intervention studies [1] determined frequency data for six quality criteria: namely, specifying the social importance of outcome variables; providing a clear description of the measure; reporting information to calculate effect sizes; administering the measures with appropriate timing; and describing the reliability and validity of outcome measures. The review provides overall compliance percentages in reporting intervention studies against the listed quality criteria. Although the authors conclude that the results of their research provide valuable information for developing and selecting outcome measures to determine the effectiveness of early numeracy interventions, an evaluation of the psychometric properties of reported measures is lacking.

Performing a psychometric review is recommended as the best starting point to support evidence-based selections of outcome measures [7]. The **CO**nsensus-based **S**tandards for the selection of health **M**easurement **IN**struments (COSMIN) group established an international consensus-based taxonomy, terminology, and definitions of measurement properties of outcome measures [8]; and published comprehensive guidelines for conducting systematic reviews on psychometric properties [9, 10]. The methodological guidelines include a checklist to assess the methodological quality of psychometric studies [11]; criteria to evaluate the psychometric quality of measures [9, 10, 12]; and a rating system to summarise psychometric evidence and grade the quality of all evidence used for the psychometric quality assessment of measures [9, 10]. Although the psychometric framework was initially developed for health-related patient-reported outcome measures, the framework has successfully been implemented in many other research areas over the past decade.

The COSMIN framework [8] distinguishes nine psychometric properties across three domains: (1) validity (i.e., the degree to which a measure measures the construct it purports to measure); (2) reliability (i.e., the degree to which a measure is free from measurement error); and (3) responsiveness (i.e., the ability of a measure to detect change over time in the construct to be measured). The domain of validity comprises five psychometric properties: 1) content validity (i.e., the degree to which the content of a measure adequately reflects the construct to be measured), 2) structural validity (i.e., the degree to which the scores are an adequate reflection of the dimensionality of the construct to be measured), 3) cross-cultural validity (i.e., the degree to which the performance of items on a translated or culturally adapted measure are an adequate reflection of the performance of the items of the original version of the measure), 4) hypothesis testing for construct validity (i.e., the degree to which scores are consistent with hypotheses on differences between relevant groups and relations to scores of other measures based on the assumption that the measure validly measures the construct to be measured), and 5) criterion validity (i.e., the degree to which scores adequately reflect a ‘gold standard’). The domain of reliability contains three psychometric properties: 1) internal consistency (i.e., the degree of the interrelatedness among items), 2) reliability (i.e., the proportion of total score variance due to true differences among respondents), and 3) measurement error (i.e., the systematic and random error of a respondent’s score not attributed to true changes in the construct measured). The third domain of responsiveness includes a single psychometric property called responsiveness and has the same definition as the domain.

When selecting a measure, content validity is the most critical psychometric property to consider [12]. Content validity shows the extent to which the content of a measure is an adequate reflection of the construct being evaluated. Good content validity ensures that the measure accurately reflects the entire construct and associated skills or knowledge in a given subject area. Using measures with poor content validity will result in flawed conclusions and unreliable decision-making processes, due to inadequate representation of the core construct. Content validity comprises three aspects of a measure [12]: (1) relevance (i.e., the degree to which all items of a measure are relevant for the construct of interest within a target population and purpose of use); (2) comprehensiveness (i.e., the degree to which all key concepts of the construct are included in a measure); and (3) comprehensibility (i.e., the degree to which items of a measure are easy to understand by respondents). A measure should only be considered for use if its items are relevant, comprehensive, and comprehensible with respect to the construct of interest and target population [12]. Therefore, the COSMIN group suggests first evaluating the development and content validity studies of a measure before considering other psychometric properties. However, a measure can only be recommended for implementation in research, education, or clinical practice if it meets predefined psychometric criteria for all measurement properties.

To date, no psychometric review on early numeracy measures has been published. In line with the COSMIN guidelines, the first psychometric property to evaluate when conducting a review on measurement properties is content validity. Therefore, this systematic review aims to evaluate the content validity of all current early numeracy measures using the COSMIN framework and methodological guidelines. Those measures with high-quality evidence for sufficient content validity can be considered in future psychometric reviews to determine the remaining psychometric properties.

## Materials and methods

This systematic review was conducted and reported by following the Preferred Reporting Items for Systematic Reviews and Meta-Analyses (PRISMA) 2020 statement and checklist [13, 14] (S1 and S2 Tables) and the COSMIN methodological guidelines [9, 10]. This psychometric review consists of three consecutive steps: (1) Systematic literature searches; (2) Evaluation of the methodological quality of studies; and (3) Evaluation of content validity of included measures. Table 1 presents an overview of the consecutive steps within this psychometric review process in line with both PRISMA and COSMIN guidelines.

**Table 1:** Study design.

STEP	DESCRIPTION	METHODOLOGY	ASPECTS (FACTORS)	RATING SCALE
**STEP 1**	**Systematic Literature Search (PRISMA)**			
Step 1.1	*Formulating eligibility criteria*			
Step 1.2	*Searching literature and selecting studies*			
**STEP 2**	**Evaluation of Methodological Quality of Studies (Risk of Bias checklist)**			
Step 2.1	*Development study*	Item generation	Relevance	Very good, Adequate, Doubtful, Inadequate, Not applicable
		Cognitive Interview / Pilot Test	Comprehensiveness & Comprehensibility	
Step 2.2	*Content validity study*	Asking target population	Relevance, Comprehensiveness, Comprehensibility	
		Asking professional	Comprehensiveness & Comprehensibility	
**STEP 3**	**Evaluation of Content Validity of Measures**			
Step 3.1	*Rating the results of single studies (Criteria for good content validity)*	Development study	Relevance	Sufficient (+), Insufficient (-), Indeterminate (?)
		Content validity study	Comprehensiveness & Comprehensibility	
		Content of measure	Comprehensiveness & Comprehensibility	
Step 3.2	*Summarising the combined results of all studies per measure*	-	Relevance, Comprehensiveness, Comprehensibility	Sufficient (+), Insufficient (-), Indeterminate (?)
Step 3.3^a^	*Grading the quality of evidence on content validity (GRADE approach)*	-	Relevance (Risk of bias, Inconsistency, Indirectness)	High, Moderate, Low, Very low
			Comprehensiveness (Risk of bias, Inconsistency, Indirectness)	
			Comprehensibility (Risk of bias, Inconsistency, Indirectness)	

Consecutive steps for PRISMA [13] and COSMIN [12]

^a^ Input from steps 2.1, 2.2, and 3.2.

The first step refers to formulating eligibility criteria (Step 1.1) and conducting systematic literature searches (Step 1.2) in line with PRISMA 2020 [13, 14] to identify early numeracy measures and reports on instrument development and/or content validity. The second step focuses on the methodological quality assessment of included studies on instrument development (Step 2.1) and content validity (Step 2.2), using the COSMIN Risk of Bias checklist [11]. The third step includes rating single study results against the criteria for good content validity (Step 3.1), summarising all results per measure (Step 3.2), and grading the quality of evidence on content validity (Step 3.3) [9].

### Systematic literature searches (step 1)

#### Information sources

Systematic literature searches were conducted in January 2024 across the following five databases to identify studies: CINAHL, Embase, Eric, PsycINFO, and PubMed. Next, reference lists of eligible articles were checked to identify additional studies. The publisher websites of Pearson, PRO-ED and Western Psychological Services were searched for additional measures and manuals that had not yet been identified by the electronic database searches and reference checking. For measures that were not freely available, the instrument developers were contacted by e-mail to gain access to the original measures.

#### Search strategies

Search strategies were performed in all five electronic databases, combining terms related to numeracy and psychometrics using both subject headings and free text terms. The full search strategies are reported in Table 2.

**Table 2:** Search strategies.

Literature Database	Search strategies
**Cinahl**	(TI (Numerac* or arithmetic* OR ((early or emergent) AND math*)) OR AB (Numerac* or arithmetic* OR ((early or emergent) AND math*))) AND ((MH "Psychometrics") OR (MH "Measurement Issues and Assessments") OR (MH "Validity") OR (MH "Predictive Validity") OR (MH "Reliability and Validity") OR (MH "Internal Validity") OR (MH "Face Validity") OR (MH "External Validity") OR (MH "Discriminant Validity") OR (MH "Criterion-Related Validity") OR (MH "Consensual Validity") OR (MH "Concurrent Validity") OR (MH "Qualitative Validity") OR (MH "Construct Validity") OR (MH "Content Validity") OR (MH "Instrument Validation") OR (MH "Validation Studies") OR (MH "Test-Retest Reliability") OR (MH "Sensitivity and Specificity") OR (MH "Reproducibility of Results") OR (MH "Reliability") OR (MH "Intrarater Reliability") OR (MH "Interrater Reliability") OR (MH "Measurement Error") OR (MH "Bias (Research)") OR (MH "Selection Bias") OR (MH "Sampling Bias") OR (MH "Precision") OR (MH "Sample Size Determination") OR (MH "Repeated Measures") OR (Psychometric* or reliabilit* or validit* or reproducibility or bias))
**Embase**	((Numerac* or arithmetic* OR ((early or emergent) AND math*)).ti. OR (Numerac* or arithmetic* OR ((early or emergent) AND math*)).ab.) AND ((psychometry/ or validity/ or reliability/ or measurement error/ or measurement precision/ or measurement repeatability/ or error/ or statistical bias/ or test retest reliability/ or intrarater reliability/ or interrater reliability/ or accuracy/ or criterion validity/ or internal validity/ or face validity/ or external validity/ or discriminant validity/ or concurrent validity/ or qualitative validity/ or construct validity/ or content validity/) OR (Psychometric* or reliabilit* or validit* or reproducibility or bias))
**Eric**	(ti(Numerac* or arithmetic* OR ((early or emergent) AND math*)) OR ab(Numerac* or arithmetic* OR ((early or emergent) AND math*))) AND ((MAINSUBJECT.EXACT("Psychometrics") OR MAINSUBJECT.EXACT("Validity") OR MAINSUBJECT.EXACT("Test Validity") OR MAINSUBJECT.EXACT("Predictive Validity") OR MAINSUBJECT.EXACT("Content Validity") OR MAINSUBJECT.EXACT("Construct Validity") OR MAINSUBJECT.EXACT("Interrater Reliability") OR MAINSUBJECT.EXACT("Reliability") OR MAINSUBJECT.EXACT("Test Reliability") OR MAINSUBJECT.EXACT("Error of Measurement") OR MAINSUBJECT.EXACT("Racial Bias") OR MAINSUBJECT.EXACT("Textbook Bias") OR MAINSUBJECT.EXACT("Test Bias") OR MAINSUBJECT.EXACT("Gender Bias") OR MAINSUBJECT.EXACT("Bias") OR MAINSUBJECT.EXACT("Social Bias") OR MAINSUBJECT.EXACT("Statistical Bias") OR MAINSUBJECT.EXACT("Accuracy") OR MAINSUBJECT.EXACT("Discriminant Analysis")) OR (Psychometric* or reliabilit* or validit* or reproducibility or bias))
**PsycINFO**	((Numerac* or arithmetic* OR ((early or emergent) AND math*)).ti. OR (Numerac* or arithmetic* OR ((early or emergent) AND math*)).ab.) AND ((Psychometrics/ OR Statistical Validity/ OR Test Validity/ OR Statistical Reliability/ OR Test Reliability/ OR Error of Measurement/ OR Errors/ OR Response Bias/ OR Interrater Reliability/ OR Repeated Measures/) OR (Psychometric* or reliabilit* or validit* or reproducibility or bias))
**PubMed**	(Numerac*[Title/Abstract] or arithmetic*[Title/Abstract] OR ((early[Title/Abstract] or emergent[Title/Abstract]) AND math*[Title/Abstract])) AND (("Psychometrics"[Mesh] or "Reproducibility of Results"[Mesh] or "Validation Studies as Topic"[Mesh] or "Bias"[Mesh] or "Observer Variation"[Mesh] or "Selection Bias"[Mesh] or "Diagnostic Errors"[Mesh] or "Dimensional Measurement Accuracy"[Mesh] or “Predictive Value of Tests"[Mesh] or "Discriminant Analysis"[Mesh]) OR (psychometric* OR reliabilit* OR validit* OR reproducibilit* OR bias))

Search strategies per literature database

#### Eligibility criteria (step 1.1)

The following inclusion and exclusion criteria were used to identify eligible measures: (1) measures assessed numeracy; (2) at least one subscale or a minimum of 50% of the total number of items of a measure referred to numeracy; (3) measures targeted children up to eight years of age; (4) measures were developed in any language, but published in English; (5) measures were published in 1995 or later; (6) only the latest version of a measure was included; and (7) psychometric data were available on measure dimensionality (i.e., the structure or dimensionality of the measure was tested by factor analysis [Classic Test Theory] and/or Rasch analysis [Item Response Theory]). Psychometric studies were eligible if: (1) studies reported on the content validity of eligible measures as defined in the COSMIN taxonomy [8]; and (2) studies were published in English. Studies on content validity could be original journal articles, manuals, or book chapters. Studies could report on the development of a measure and/or the relevance, comprehensiveness, or comprehensibility of the content of the measures [8].

#### Data collection process

Data points across all psychometric studies and numeracy measures were extracted using comprehensive data extraction forms of the COSMIN methodology for assessing the content validity of measures [12]. In line with the COSMIN recommendations, single study results were rated against the criteria for good content validity; results of studies per measure were summarised; and the quality of evidence on content validity was graded. Characteristics of the included measures for the assessment of numeracy were extrapolated and synthesised against the following categories: full name of measure and acronym, target population, number of subscales (assessment tasks) and the total number of items, ways of administration, completion duration, and response options.

#### Data, items, risk of bias, and synthesis of results

Two independent raters reviewed all titles and abstracts for eligibility. Both reviewers also assessed the original reports for eligibility. If both reviewers could not agree, a third reviewer was consulted to achieve 100% consensus. The risk of bias per individual study was assessed using the COSMIN Risk of Bias checklist [11]. Two independent researchers (SY and J-HK) assessed the methodological study quality (COSMIN Risk of Bias checklist), after which consensus was reached with the involvement of a third reviewer (RS and RC) when necessary. The same procedure was followed when assessing the content validity of measures; two raters (SY and J-HK) evaluated all measures, after which disagreements were resolved by group consensus inviting additional reviewers (RS and RC). As none of the reviewers had formal or informal affiliations with any of the authors of the included studies and measures, no evident bias in article selection or methodological study quality rating was present.

### Evaluation of methodological quality of studies (step 2)

The methodological quality of the included studies on instrument development (Step 2.1) and content validity (Step 2.2) was assessed using the COSMIN Risk of Bias checklist [11]. Studies describing the development of a measure were assessed using the first 35 items of the COSMIN checklist, which consists of the following two parts: 1) quality ratings of the measure design (item generation) to ensure relevance, and 2) quality ratings of cognitive interviews or other pilot tests to evaluate comprehensiveness and comprehensibility of a draft measure [11]. Next, the quality of included content validity studies was assessed using another 31-item checklist involving two sets of items. The first set of items assessed studies that asked representatives from the target population (e.g., patients in patient-reported outcome measures or children in early numeracy measures) about the relevance, comprehensiveness, and comprehensibility of test items; and the second set of items assessed studies that asked experts or professionals (e.g., teachers) about the relevance and comprehensiveness of test items [9]. Total ratings were determined separately for each of the three aspects of content validity (i.e., relevance, comprehensiveness, and comprehensibility), both checklist parts for developmental studies (i.e., ‘instrument design’ and ‘cognitive interview or pilot test’), and both types of content validity studies (i.e., ‘asking children’ and ‘asking professionals’) [11].

Each checklist item was scored on a 4-point rating scale (1 = inadequate, 2 = doubtful, 3 = adequate, and 4 = very good). Total ratings for relevance, comprehensiveness, and comprehensibility were calculated using the following equation [15]:

$$\text{Total}\;\text{(*Relevance*Comprehensiveness*Comprehensibility)}\;\text{rating}=\frac{\text{Total}\;\text{score}\;-\;\text{minimal}\;\text{score}\;\text{possible}}{\text{Maximum}\;\text{score}\;\text{possible}-\text{minimum}\;\text{score}\;\text{possible}}\times 100$$

Percentage scores were preferred over the worst score count method as suggested by the COSMIN guidelines, which takes the lowest ratings among any of the checklist items as the final score [11]. This decision was taken as determining total scores of methodological study quality based on the lowest rating of single items impedes the detection of nuanced differences in methodological quality between studies [16]. Next, the total percentage scores were categorised as follows: inadequate (from 0% to 25%), doubtful (from 25.1% to 50%), adequate (from 50.1% to 75%), and very good (from 75.1% to 100%). The methodological quality was rated by two reviewers (SY and J-HK) independently, whereafter consensus ratings were determined between both reviewers. The interrater reliability between reviewers was calculated using weighted *Ƙ* [17].

After completing the assessment of methodological quality on the included instrument development and content validity studies, data were extracted from the included studies and measures against the following categories: (1) study characteristics (i.e., study purpose and study population); (2) measure characteristics (i.e., measure names and acronyms, measured constructs, targeted population, number of scales and subscales, number of items, and response options); and (3) study results on all three aspects of content validity (relevance, comprehensiveness, and comprehensibility). Data were extracted by one reviewer (CW and AH) and rechecked for accuracy by another reviewer (SWG).

### Evaluation of content validity of measures (step 3)

Each of the three aspects of content validity (relevance, comprehensiveness, and comprehensibility) was assessed separately in three sequential steps (Steps 3.1, 3.2, and 3.3). Similar to methodological quality ratings, two reviewers conducted content validity ratings independently, after which discrepancies were resolved to reach consensus. Both reviewers had considerable expertise in psychometrics and the COSMIN methodology.

#### Rating the result of single studies (step 3.1)

The results for each instrument development study, content validity study, and content of the measure itself, were rated separately. Ratings were based on the qualitative or quantitative data obtained by asking children and/or professionals about measures’ content validity, using ten predefined criteria on relevance (5 criteria), comprehensiveness (1 criterion), and comprehensibility (4 criteria) [9]. The same ten criteria were used when rating the content of the original measure itself (items, response options, and recall period) based on the reviewers’ subjective judgement. Ratings for each source of evidence on content validity were sufficient (≥ 85% of the measure items meet the criterion: +), insufficient (< 85% of the measure items meet the criterion: −), or indeterminate (lack of evidence to determine the quality or inadequate methodological quality of studies:?). Further details on the predefined criteria and how to apply them can be found in the user manual on COSMIN methodology for assessing content validity [12].

#### Summarising the results of all studies per measure (step 3.2)

All results from available studies on development and content validity per measure; and the reviewers’ ratings on content of the measure were qualitatively summarised into overall ratings for relevance, comprehensiveness, and comprehensibility of the measure [9, 12]. While the previous step (Step 3.1) focused on single studies, Step 3.2 focused on a specific measure. An overall rating of sufficient (+), insufficient (−), inconsistent (±), or indeterminate (?) was calculated for relevance, comprehensiveness, and comprehensibility for each measure [9, 12]. For example, if all relevance scores of development studies, content validity studies, and content of the measure (reviewers’ ratings) were rated as sufficient, insufficient, or indeterminate, the overall relevance rating became, respectively, sufficient (+), insufficient (−), or indeterminate (?). However, if one or more of these three scores was inconsistent with the other two scores, the overall rating became inconsistent (+). An exception to this rule was when the scores of both development and content validity studies, were all indeterminate and inconsistent with the reviewers’ ratings on the content of the measure; in this instance, the overall rating may be determined by the reviewers’ rating solely. Further details on rating overall relevance, comprehensiveness, and comprehensibility are described in the COSMIN user manual for assessing content validity [12].

#### Grading the quality of evidence on content validity (step 3.3)

The quality of the evidence (i.e., the total body of evidence used for overall ratings on relevance, comprehensiveness, and comprehensibility of a measure) was graded using a modified Grading of Recommendations Assessment, Development and Evaluation (GRADE) approach [9, 18]. The starting point of this evidence quality rating is based on the assumption that the overall rating is of high quality, whereafter the GRADE approach is used to downgrade the level of evidence when concerns about the quality of evidence are identified. If serious or very serious risk of bias (i.e., limitations in the methodological quality of studies), inconsistency (i.e., unexplained heterogeneity in results of studies), and/or indirectness (i.e., evidence from different populations than the target population of interest in the review) are present, ratings are downgraded one or more levels (to moderate, low, or very low) [9, 12]. The quality of evidence was not graded if overall ratings were indeterminate (?) due to lack of evidence. More detailed information about grading the quality of evidence can be found in the COSMIN user manual for content validity [12].

## Results

### Systematic literature searches

**Fig 1:**
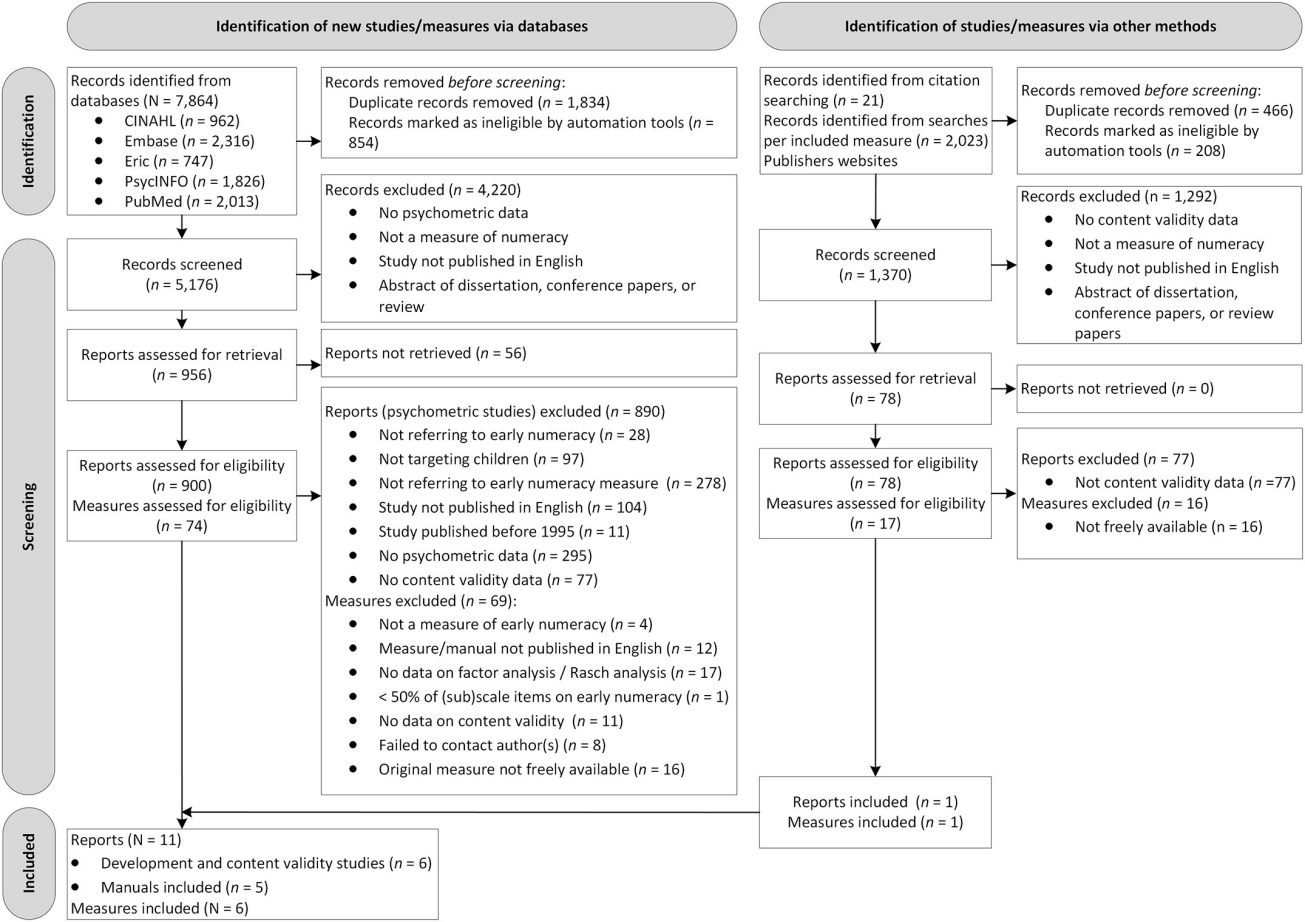
PRISMA flow diagram. EarlyNumeracy”. The reviewing process according to PRISMA [13, 14] **.

The final literature searches to identify early numeracy measures and related psychometric studies were in January 2024. In total, these original searches identified 7,864 records from the following five electronic databases: CINAHL (*n* = 962), Embase (*n* = 2,316), Eric (*n* = 747), PsycINFO (*n* = 1,826) and PubMed (*n* = 2,013). Next, to identify any missing studies from the previous searches, additional literature searches using full names and acronyms of included measures were conducted (January 2024), retrieving an additional 2,023 records. In total, 978 papers (original searches: *n* = 900; additional searches: *n* = 78) and 91 measures (original searches: *n* = 74; additional searches and publishers’ websites: *n* = 17) were assessed for eligibility. S3 Table presents a list of excluded measures and reasons for exclusion. In total, six measures, five manuals, and six development and content validity studies were included in this review. Fig 1 shows the flow diagram of the reviewing process according to PRISMA.

### Characteristics of included studies and measures

Descriptions of the instrument development or content validity studies of the included measures are presented in S4 Table (data extractors: CW and SWG; data extraction period: Feb 2024). Table 3 provides a summary of the characteristics of the six included measures against the following descriptors (data extractors: CW, AH and SWG; data extraction period: Feb 2024): acronyms and full names of measures, target population (grade level and age group), number of subscales related to early numeracy (assessment tasks and description) and the total number of items, ways of administration, completion duration, and response options. The total number of early numeracy assessment tasks varied between 2 and 12, with the total number of items ranging from 5 to 76. Completion time was not always reported (IDELA) [19, 20] or was dependent on the number of assessment tasks evaluated (ELOM) [21]. For the other measures, the estimated time to complete ranged between less than 5 minutes, as with the CPM [22] and PENS-B [23], and about 20 minutes as with DK-TEAM [24] and Core EGMA [25]. Overall, most measures used individual administration (e.g., interview by a trained assessor) with a wide range of distinct scoring systems.

**Table 3:** Measure characteristics.

Acronym Measure Name (Authors, date, country)	Grade level Age group Target population	Assessment tasks ^a^ (Items *n*): Task description Total number of assessment tasks ^a^ (Total number of items) ^a^	Administration Duration Scoring
CPM Circle Progress Monitoring (Assel et al., 2020, United States of America) [22]	Pre-kindergarten 3–4 years	**Rote counting** (*n* = 1 item): highest number counted **Shape naming** (*n* = 5 items): circle, square, triangle, rectangle, oval **Number naming** (*n* = 2 items): points to 4, 7 **Number recognition** (*n* = 5 items): identifies 2, 5, 8, 13, 16 **Shape discrimination** (*n* = 6 items): points to, points to last, and points to different triangle/square **Counting** (*n* = 5 items): cardinal value 3, 5, 7, 10, 15 **Operations** (*n* = 3 items): 3–1 (drinks), 2+1 (trucks), 5–2 (butterflies). **N**_**Tasks**_ = 7 (**N**_**Items**_ = 27)	**Administration:** one-to-one electronic administration with a teacher. **Duration:** 4–6mins. **Scoring:** % items correct. Total score range: 0–100%.
Core EGMA Core Early Grade Mathematics Assessment (Platas et al., 2014, United States of America) [25]	Grade 1–3 6–9 years	**Number identification** (*n* = 20 items): correctly read aloud the most accurate 1–3 digit numbers of increasing complexity; timed, 60secs **Number discrimination** (*n* = 10 items): identify a higher number from 10 pairs of 1–3 digit numbers; untimed, stop after 4 successive errors **Missing numbers** (*n* = 10 items): in 4 horizontally aligned boxes, 3 contain numbers following a pattern with 1 box empty: student must identify what number should be in the empty box; patterns vary; 1–3 digit numbers; untimed, stop after 4 successive errors **Addition & subtraction level 1** (*n* = 20 items): sums of increasing difficulty; no addends >10; no sum >19 (timed, 60secs) **Addition & subtraction level 2** (*n* = 10 items): not administered where students score 0 in level 1; sums of increasing difficulties; no sums >70; untimed, stop after 4 successive errors **Word problems** (*n* = 6 items): orally presented real-world addition/subtraction problems: change, result unknown; combine, result unknown; compare, change unknown; change, start unknown; sharing; multiplicative. Untimed, stop after 4 successive errors. **N**_**Tasks**_ = 6 (**N**_**Items**_ = 76)	**Administration:** Oral assessment, individually administered to students by trained assessors. Assessor training and observation checklist required. EGMA data can be gathered through paper instruments or on mobile devices, such as tablets, using the RTI-developed Tangerine^®^ software (see www.tangerinecentral.org). **Duration:** 25mins. **Scoring:** Results reported per subtest; reported as raw scores for untimed tests; timed subtests reported as a rate, i.e., correct answers per minute. Total score (if requested)–as scales differ for each subtest, aggregated subtest scores should be used with care. Timed subtests—the average rate of correct responses per minute (Total score range: 0 –total subtest -items/min.); untimed subtests—scores can be aggregated by determining the average of the proportion of items correctly answered (Total score range: 0–100%)..
ELOM Early Learning Outcomes Measure (Dawes et al., 2016, South Africa) [21]	Grade R—South African pre-school 50–59 months; 60–69 months	**Emergent numeracy and mathematics (domain)** **Number concepts** (*n* = 2 items): counts 3, 8, 15 objects; simple addition and subtraction arithmetic using picture card stimulus **Symbol, shape, size and space understanding** (*n* = 3): group stars and circles by colour and shape; identify objects in a picture that are above/under, in front of, on the side; identify biggest, smallest, longest, and shortest from pictures. **N**_**Tasks**_ = 2 (**N**_**Items**_ = 5)	**Administration:** Direct assessment by trained assessor. Equipment required for administration is provided in the ELOM Direct Assessment Kit list. All other components required are available for download from Innovation Edge ELOM website. **Duration:** 45 mins (*when all indicator measures are combined; numeracy domain is one of six; individual domain durations NR*) **Scoring**: 1 = correct; 0 = incorrect. Tablets/android mobile phones should preferably be used for scoring. Total score range: 0–5.
IDELA International Development and Early Learning Assessment (Pisani et al., 2015, 2018, United Kingdom) [19, 20]	Preschool 3.5–6.5 years All populations (adaption instructions for children with disabilities provided within administration manual)	**Emerging numeracy (domain)** **Comparison by size & length** (*n* = 4 items): identify biggest/smallest/longest/shortest circle/stick **Sorting & classification by shape & colour** (*n* = 2 items): child sorts cards by two criterion **Shape identification** (*n* = 5 items): identify circle, rectangle, triangle, square; identify “something” in the environment shaped like a circle **Number identification** (*n* = 1 item): Identify number from 1–20 –pause >5sec, move on to next **One-to-one correspondence** (*n* = 3 items): give assessor 3, 8, 15 objects from 20 (i.e., beans)–if the child cannot provide 3 or 8, stop; **Addition & subtraction** (*n* = 3 items): using objects from the previous item (e.g., beans) and picture cards, add/subtract 3+2, 2+2, 3–1 **Puzzle completion** (*n* = 1 item): 4 or 6-piece puzzle. **N**_**Tasks**_ = 7 (**N**_**Items**_ = 19)	**Administration:** one-to-one with assessor and child. Instruction manual for administration details objectives, instructions, adaptation instructions, scoring instructions. Require: manual, pencil, timing device (mobile/watch), stimuli (i.e., picture cards). **Duration:** estimated duration NR. No time limit to complete the assessment. Some items are to be scored at 2mins (only applicable to puzzle completion in the Emerging numeracy domain. **Scoring:** 1 = correct; 0 = incorrect/do not know; 999 = refused/skipped; continuous score items (i.e., number of correctly placed jigsaw puzzles, maximum 10). Total score range: 0–19.
PENS-B Preschool Early Numeracy Screener—Brief Version^b^ (Purpura et al., 2015, United States of America) [26, 27]	Preschool 3–5years English speakers	**One-to-one counting** (*n* = 3): count dots **Counting a subset** (*n* = 2): count a given number of pictures from a larger set **Set comparison-most** (*n* = 2): identify the highest number of dots from 4 sets of dots **Numeral identification** (*n* = 1): correctly identify numbers visually **Set-to-numerals** (*n* = 3): connect the number to the correct number of dots **Number order** (*n* = 2): identify the number before/after the given number **Number comparison-most** (*n* = 1): identify the highest number from 4 numbers **Relative size** (*n* = 2): identify the number closest to a given number **Story problems** (*n* = 3): simple addition/subtraction problems presented as stories **Number combinations** (*n* = 4): simple additions **Ordinality** (*n* = 1): identify 8th object **N**_**Tasks**_ = 11 (**N**_**Items**_ = 24)	**Administration:** appropriately trained examiner; one-to-one assessment with manual and record form. Three components required—Examiner’s Manual, Picture Book, Examiner Record Form. **Duration:** 5mins or less (child age/ability dependent) **Scoring:** Item 1 –up to 5 points dependent on the highest number counted; items 2–24 1 = correct / 0 = incorrect. Total raw score converted to a standard score, Early Numeracy Index. Total score range: 0–24.
DK-TEAM Tools for Early Assessment in Math—Danish version (Sjoe et al., 2019, Denmark) [24]	Preschool 3–6 years Typically developing and children at risk for delay	**[Domain: Geometry** (*n* = 7)] **Shape recognition** (*n* = 3): place an object in a square; use straws to make a triangle; identify sides of a geometric shape. **Comparing shapes** (*n* = 3): match a/typical shapes **Patterns and pre-algebraic thinking** (*n* = 1): “one block is missing, can you fix this pattern?” **[Domain: Numeracy** (*n* = 12)**]** **Counting** (*n* = 6): highest number counting; count/hide 4 or 8 objects; “what comes before 8?” **Comparing and ordering numbers** (*n* = 3): identify larger quantities from 2 options; put numbers of objects (cards/muffins) in order **Numerals** (*n* = 1): match the numeral to set **Composing numbers** (*n* = 2): count 4 or 8, hide 2, ask how many? **N**_**Tasks**_ = 7 (**N**_**Items**_ = 19)	**Administration:** administered by childcare workers provided with an information manual; one-to-one with child; responses collected in an online format on a tablet. **Duration: a**verage completion time 18mins. **Scoring:** 0 = incorrect; up to 4 = correct. Automatically scored on “Ramboll Results” online system. Total score range: 0–24 (Geometry: 0–8; Numeracy: 0–16).

Characteristics of the included measures for the assessment of early numeracy (alphabetical order)

*Note*. mins = minutes; NR = Not reported

^a^ Only assessment tasks related to early numeracy reported.

^b^ PENS-B is a revised version of PENS (Preschool Early Numeracy Screener). PENS includes 25 items, showing large overlap with PENS-B.

### Methodological quality of development and content validity studies

The methodological quality of the 11 included studies (including the manuals) on instrument development and content validity was assessed using the COSMIN checklist [11] (data extractors: SY and J-HK; data extraction period: March 2024). Eight content validity studies (*n* = 8) overlapped with development studies (*n* = 11). Table 4 presents an overview of the methodological quality ratings of the included measures’ development and content validity studies. Of all ratings on relevance, comprehensiveness, and comprehensibility based on the development and content validity studies, 46% of ratings (22/48) were categorised as not reported, 12% (6/48) as adequate, 29% (14/48) as indeterminate, and 12% (6/48) as doubtful.

**Table 4:** Methodological quality assessment.

Measure	Development Study Quality ^a^			Content Validity Study Quality ^a^				
	Item generation ^b^	Cognitive interview ^b^		Asking children ^b^			Asking professionals ^b^	
	Relevance	Comprehen-siveness	Comprehen-sibility	Relevance	Comprehen-siveness	Comprehen-sibility	Relevance	Comprehen-siveness
**CPM** [22]	Doubtful (25.5%)	Inadequate (4.8%)	Inadequate (4.8%)	NR	NR	NR	Adequate (66.7%)	Adequate (66.7%)
**Core EGMA** [25, 28, 29]	Doubtful (25.5%)	Inadequate (2.3%)	Inadequate (2.3%)	NR	NR	NR	Adequate (66.7%)	Adequate (66.7%)
**ELOM** [21]	Inadequate (17.0%)	Inadequate (2.4%)	Inadequate (2.4%)	NR	NR	NR	Adequate (60.0%)	NR
**IDELA** [19, 20]	Doubtful (25.5%)	Inadequate (2.4%)	Inadequate (2.4%)	NR	NR	NR	Doubtful (33.3%)	NR
**PENS** [23, 26, 27]	Doubtful (25.5%)	Inadequate (2.4%)	Inadequate (2.4%)	NR	NR	NR	Inadequate (11.1%)	NR
**DK-TEAM (Danish version)** [24]	Doubtful (25.5%)	Inadequate (2.4%)	Inadequate (4.8%)	NR	NR	NR	NR	Adequate (60.0%)

Methodological quality assessment of development and content validity studies of the included measures

^a^ The methodological quality per development and content study was rated using the COSMIN checklist [11] as very good, adequate, doubtful, and inadequate. The overall methodological quality per study was presented as a percentage of the ratings [16]: Inadequate = 0–25%, Doubtful = 25.1–50.0%, Adequate = 50.1–75%, Very good = 75.1–100.0%; NR = Not Reported.

^b^ The methodological quality was rated in the three aspects of content validity: (1) the relevance (all items of a measure are relevant for the construct of interest); (2) comprehensiveness (the degree to which all key concepts of the construct are included in a measure); and (3) comprehensibility (the degree to which items of a measure are easy to understand by respondents). The methodological quality of development studies was rated in two parts: (1) concept elicitation (relevance) and (2) cognitive interview (comprehensiveness and comprehensibility). The methodological quality of content validity studies was rated in two different study categories asking children or professionals about the relevance, comprehensiveness, and comprehensibility.

*Note*. CPM = Circle Progress Monitoring; Core EGMA = Core Early Grade Mathematics Assessment; ELOM = Early Learning Outcomes Measure; IDELA = International Development and Early Learning Assessment; PENS-B = Preschool Early Numeracy Screener—Brief Screener; DK-TEAM = Tools for Early Assessment in Math—Danish version.

All instrument development quality ratings for comprehensiveness and comprehensibility were classified as inadequate, while all relevance ratings were classified as doubtful, except for one study that was rated as inadequate. None of the content validity studies involved children in determining relevance, comprehensiveness, and comprehensibility. Professionals were involved in determining the relevance of items for five measures, of which three ratings were classified as adequate, one rating as doubtful and one as inadequate. Professionals provided feedback on the comprehensiveness of three measures, which were rated as adequate. The interrater reliability for study quality assessment between both reviewers was excellent [17]: weighted Ƙ = .90 (95% CI [.82,.97]).

### Content validity of measures

Table 5 presents the overall quality of content validity for relevance, comprehensiveness, and comprehensibility, respectively, and evidence quality per measure (i.e., the confidence level for the overall quality rating of content validity). None of the measures received sufficient overall quality of content validity ratings across *all three aspects* of content validity (i.e., relevance, comprehensiveness, and comprehensibility). However, all measures scored sufficient ratings for both relevance and comprehensibility, but ratings for comprehensiveness were either insufficient or inconsistent (Core EGMA [25] and DK-TEAM, Danish version [24]). Limited high-quality evidence supporting overall ratings on content validity was available. Two measures, the CPM [22] and Core EGMA [25], provided high quality evidence for relevance. Of note, 61% (11/18) of all evidence quality ratings for content validity were rated as very low. In particular, ratings of the quality of evidence for comprehensibility were classified as very low for all six measures.

**Table 5:** Overall quality of content validity and evidence.

Measure	Relevance		Comprehensiveness		Comprehensibility	
	Overall quality of content validity ^a^	Quality of evidence ^b^	Overall quality of content validity ^a^	Quality of evidence ^b^	Overall quality of content validity ^a^	Quality of evidence ^b^
**CPM (Assel et al., 2020)**	+	High	–	Very Low	+	Very Low
**Core EGMA (Platas et al., 2014)**	+	High	±	Moderate	+	Very Low
**ELOM (Dawes et al., 2016)**	+	Moderate	–	Very Low	+	Very Low
**IDELA (Pisani et al., 2015, 2018)**	+	Moderate	–	Very Low	+	Very Low
**PENS-B (Purpura et al., 2015)**	+	Low	–	Very Low	+	Very Low
**DK-TEAM (Danish version) (Sjoe et al., 2019)**	+	Very Low	±	Moderate	+	Very Low

Overall quality of content validity and evidence quality per measure.

*Notes*. CPM = Circle Progress Monitoring; Core EGMA = Core Early Grade Mathematics Assessment; ELOM = Early Learning Outcomes Measure; IDELA = International Development and Early Learning Assessment; PENS-B = Preschool Early Numeracy Screener—Brief Version; DK-TEAM = Tools for Early Assessment in Math—Danish version.

^a^ The overall quality of content validity (relevance, comprehensiveness, and comprehensibility) was determined by qualitatively summarising all ratings on content validity per study of each instrument and reviewers’ ratings on the content of the instrument itself [9]: + = sufficient rating;? = indeterminate rating;– = insufficient rating; ± = inconsistent rating.

^b^ The quality of evidence (confidence level for the overall quality rating of content validity) was rated using a modified Grading of Recommendations Assessment, Development and Evaluation approach (9); high = high level of confidence; moderate = moderate level of confidence; low = low level of confidence; very low = very low level of confidence.

## Discussion

This systematic review aimed to determine the quality of content validity of current early numeracy measures. A total of six measures met all inclusion criteria resulting in 11 corresponding instrument development and content validity studies (including five manuals). The COSMIN ratings demonstrated lack of high-quality evidence. Furthermore, none of the measures received high-quality ratings for *all three* aspects of content validity (i.e., relevance, comprehensiveness, and comprehensibility). Therefore, the use of the included measures cannot be supported in terms of the quality of content validity.

### Measures

Since early numeracy is an umbrella term for a multidimensional phenomenon encompassing several related skills [1], all measures comprised multiple domains associated with early numeracy, reflecting the multidimensional character of the construct of early numeracy. However, while the included six measures showed some commonalities in aspects of early numeracy being targeted, there was also considerable variability. This finding is unsurprising given that several models of early numeracy have been published [5], yet there is no international consensus to reconcile the differences between existing models. The variability in the content of included measures reflects this lack of consensus. It highlights the need for future research to evaluate the structure of early numeracy in a more systematic and coordinated way to increase comparability and coherence across studies [5].

Apart from which early numeracy aspects are covered in a measure, measure selection may also depend on the purpose of a measure. Measures are used with different goals in mind. For example, as an initial screening, diagnosing an impairment, identifying focus areas for intervention, facilitating decision-making about service delivery, or outcome measurement following the introduction of an intervention [30]. This review excluded screening tools since, by default, screening measures are designed to identify those at risk of numeracy difficulties, after which further assessment may be required.

### Instrument development and content validity studies

Target populations per measure differed in age. In general, the expected target population for early numeracy measures will be up to eight years of age. The COSMIN guidelines consider the involvement of the target population as an essential step in the instrument development process when constructing a measure [9]. In line with these guidelines, conducting cognitive interviews with future users of these measures, including educators and children at risk of difficulties in early numeracy skills, is considered an essential step in the instrument development process. Furthermore, when conducting content validity studies, target populations (i.e., children and professionals) should be involved and provide feedback on all three aspects of content validity (i.e., reliability, comprehensiveness, and comprehensibility). However, considering the young age of target populations in early numeracy measurement, it is expected to retrieve limited information from the literature about asking for children’s feedback on content validity aspects. Therefore, mainly focusing on professionals’ opinions about relevance and comprehensiveness might be considered a more appropriate approach when targeting younger populations. However, despite the impact of excluding young target populations on the methodological quality ratings, many measures received poor scores due to incomplete reporting or not providing data at all.

### Synthesis of evidence on content validity

According to the COSMIN guidelines, content validity is the first and most critical psychometric property to be considered when selecting measures [9]. However, given the inadequate quality of evidence, measure selection based on content validity is seriously hampered. The results on the methodological quality of development and content validity studies were poor, given that a majority of ratings were scored as ‘not reported’, ‘indeterminate’, or ‘doubtful’. As a result, many overall ratings on content validity aspects were based solely on reviewers’ subjective opinions about the content of the included measures. Overall, the lack of evidence for content validity and the use of inappropriate methodological approaches were identified in both instrument development and content validity studies. Consequently, evidence of comprehensibility and comprehensiveness were categorised as very low. Evidence of relevance of content validity was scored higher; two measures showed high levels of evidence (CPM and Core EGMA), and two measures had moderate levels of evidence (ELOM and IDELA). However, based on the current data retrieved in this review, only very preliminary conclusions can be made since no conclusive evidence is available for any of the included measures. Even so, because no high-quality evidence for insufficient relevance, comprehensiveness, or comprehensibility for any of the measures was determined, all included measures may have the potential to be used in terms of content validity.

### Limitations

This systematic review has some limitations. Firstly, only measures and psychometric studies published in English were considered. Thus, findings on the content validity of early numeracy measures developed in languages other than English have been excluded. Secondly, despite contacting the developers of measures, several measures could not be retrieved from the authors or literature. As such, these measures were excluded from this review as no overall ratings on content validity could be determined. Limited access to identified measures in the domain of early numeracy may have negatively impacted the completeness of the current overview of content validity of early numeracy measures. Publishers and instrument developers should consider providing researchers access to their measures for future psychometric evaluations, thereby supporting educators and other potential users of these measures in their selection of psychometrically robust measures to identify children at risk of early numeracy difficulties.

Moreover, this review has its strengths. Most importantly, this review followed the COSMIN methodological guidelines as well as the PRISMA 2020 statement and checklist, providing methodological rigour to this review.

## Conclusion

Six measures of early numeracy were retrieved from the literature. None of these measures can be recommended for use in clinical practice, education, or research due to the lack of high-quality evidence on content validity. However, since no high-quality evidence was found to indicate insufficient content validity, there are no firm recommendations against using the included measures. Future studies should evaluate the remaining psychometric properties of the included measures to identify the most robust measures in terms of validity, reliability, and responsiveness.

## Supporting information

S1 Table PRISMA 2020 abstracts checklist. (DOCX)

S2 Table PRISMA 2020 checklist. (DOCX)

S3 Table Excluded measures and reasons for exclusion. (DOCX)

S4 Table Study details. Description of the development and content validity studies on included measures.
(DOCX)
