# Supplementary material for: Content validity of measures in early numeracy in children up to eight years: A COSMIN systematic review
Source: PLoS One. 2024 Sep 19;19(9):e0308874. doi: 10.1371/journal.pone.0308874 (PMC11412641; doi:10.1371/journal.pone.0308874)
Supplement: S3 Table — (DOCX) [file pone.0308874.s003.docx]

**S3 Table. Excluded measures.** With reasons for exclusion.

| No | Measure *(alphabetical order)* | Acronym | Reason for exclusion |
| --- | --- | --- | --- |
|  | Academic Achievement Battery (Messer, 2014) [1] | AAB | Original measure/items not freely available |
|  | Acadience Math Assessment (Wheeler, et al., 2019) [2] |  | No data on content validity |
|  | aimswebPlus (Pearson, 2017) [3] |  | Original measure/items not freely available |
|  | Ani Banani Math Test. (Ten Braak, et al., 2021) [4] | ABMT | No data on content validity |
|  | Approximate Symbolic Arithmetic (Gilmore, et al., 2007) [5] |  | No factor analysis or Rasch analysis |
|  | Best Start Kindergarten Assessment (Harris, et al., 2018) [6] | Best start | Failed to contact authors |
|  | Boehm Test of Basic Concepts-Third edition (Dumont & Willis, 2008) [7] | Boehm-3 | Original measure/items not freely available |
|  | Boehm Test of Basic Concepts, Preschool: Boehm 3 (Boehm, 2000) [8] | Boehm-3 Preschool | Original measure/items not freely available |
|  | Bracken Basic Concepts Scale - Third Edition: Receptive. (Bracken, 2006) [9] | BBCS-3:R | No factor analysis or Rasch analysis |
|  | Brief Number Screener. (Jordan, et al., 2010) [10] | NSB | No factor analysis or Rasch analysis |
|  | Child Math Assessment (Milburn, et al., 2019) [11] | CMA | No data on content validity |
|  | Comprehensive Research-based Early Math Ability Test (Clements, et al., 2020) [12] | CREMAT | Original measure/items not freely available |
|  | Computer-Based Assessment of School Readiness and Early Reasoning (Csapo, et al., 2014) [13] |  | Less than 50% of total number of items on early numeracy |
|  | Computerised d tasks (Greisen, et al., 2018; Greisen, 2020 [dissert.]) [14] | NUMTEST | No data on content validity |
|  | Curriculum-based measurement – Kindergarten (Seethaler & Fuchs, 2011) [15] | CBM-K | No factor analysis or Rasch analysis |
|  | Curriculum-based measures of math performance for preschool children (VanDerHeyden, et al., 2004) [16] | CBM | No factor analysis or Rasch analysis |
|  | Curriculum-based probes for Kindergarteners in reading, math, and writing readiness skills (3 Math probes) (VanDerHeyden, et al., 2001) [17] | CBM-K | No factor analysis or Rasch analysis |
|  | Detroit tests of Learning Abilities, 5^th^ edition (Hammill, McGhee & Ehrler, 2018) [18] | ***DTLA-5*** | Original measure/items not freely available |
|  | Deutscher Mathematiktest für erste Klassen (Krajewski, et al., 2002) [19] | DEMAT 1+ | Measure not published in English |
|  | Developmental Tasks for Kindergarten Readiness-2^nd^ ed (Lesiak & Lesiak, 1978) [20] | DTKR-II | Original measure/items not freely available |
|  | Diagnostic Achievement Battery-4^th^ edition (Newcomer, 2014) [21] | DAB-4 | Original measure/items not freely available |
|  | Digital Home Numeracy Practice (Alam, et al., 2023) [22] | DHNP-i | Not a measure of early numeracy |
|  | Dynamic Assessment method (Baek & Kim, 2003) [23] |  | Not a measure of early numeracy |
|  | Early Arithmetic, Reading, and Learning Indicators (DiPerna, et al., 2007) [24] | EARLI | Failed to contact authors |
|  | Early Arithmetic, Reading, and Learning Indicators Short Forms (Lei, et al., 2009) [25] | EARLI | Original measure/items not freely available |
|  | Early Mathematics Curriculum-Based Measurement (Clarke & Shinn, 2004) [26] | EM-CBM | No factor analysis or Rasch analysis |
|  | Early Mathematics Measures (Lee, et al., 2012) [27] | CBM | Original measure/items not freely available |
|  | Early Mathematics Measures (Lembke, et al., 2008) [28] |  | No factor analysis or Rasch analysis |
|  | Early Numeracy Assessment (Thomas, et al., 2023) [29] | ENA | Measure not published in English |
|  | Early Numeracy Curriculum-based Measurement (Chard et al., 2005) [30] | EN-CBM | No factor analysis or Rasch analysis |
|  | Early Numeracy Indicators (Lembke & Foegen, 2009) [31] | ENIs | No factor analysis or Rasch analysis |
|  | Early Numeracy Indicators (Conoyer, et al., 2016) [32] | ENI | No factor analysis or Rasch analysis |
|  | Early Numeracy Screener (Lopez-Pedersen, 2020) [33] |  | Measure not published in English |
|  | Early Numeracy Skill Indicators (Methe, et al., 2011) [34] | ENSI | No factor analysis or Rasch analysis |
|  | The Early Numeracy Test in Finnish (Aunio, et al., 2006) [35] | ENT-Fin | Measure not published in English |
|  | Early Numeracy Test in Flemish (Torbeyns, et al, 2002) [36] | ENT-Fle | Measure not published in English |
|  | Early Numeracy Test Romanian version form B (David, et al., 2015) [37] | ENT-Rom | Measure not published in English |
|  | Feifer Assessment of Mathematics (Feifer & Clark, 2016) [38] | FAM | Original measure/items not freely available |
|  | Group Mathematics Assessment and Diagnostic Evaluation (Williams, 2004) [39] | GMADE | Original measure/items not freely available |
|  | Indicadores de Progreso de Aprendizaje en Matemáticas / (Indicators of Basic Early Math Skills) (Jiménez & de León, 2019) [40] | IPAM | Original measure/items not freely available |
|  | Indicadores de Progreso de Aprendizaje en Matemáticas / (Indicators of Basic Early Math Skills; de León, et al., 2021) [41] | IPAM-Spa | Measure not published in English |
|  | Individual Growth and Development Indicators–Early Numeracy (Hojnoski & Floyd, 2013) [42] | IGDIs-EN | Failed to contact authors |
|  | Kaufman Survey of Early Academic and Language Skills (Kaufman, 1993) [43] | K-SEALS | Original measure/items not freely available |
|  | Kaufman Test of Educational Achievement-3 (Frame, Vidrine & Hinojosa, 2016) [44] | K-TEA-3 | Original measure/items not freely available |
|  | Kaufman Test of Educational Achievement-3, Brief (Kaufman, 2015) [45] | K-TEA-3, brief | Original measure/items not freely available |
|  | KeyMath-3 Essential Resources, Level 1 (Connolly, 2007) [46] | KeyMath 3 | Original measure/items not freely available |
|  | Laotian Numeracy Subtest (Gomez, et al., 2022) [47] | MELQO | Measure not published in English |
|  | The little math quiz (Arendasy, et al., 2005) [48] |  | Failed to contact authors |
|  | Math Essential Skill Screener–Elementary Version (Erford, et al., 1998) [49] | MESS-E | No data on content validity |
|  | Mathematics assessment (Kindergarten; 1^st^ grade; 2^nd^ grade) (Lee, & Lembke, 2016) [50] | CBM | Original measure/items not freely available |
|  | Mathematics Fluency and Calculation Tests (Reynolds, Voress & Kamphaus, 2015) [51] | MFaCTS | Original measure/items not freely available |
|  | Mathematik- und Rechenkonzepte im Vorschulalter – Diagnose (Ricken, et al., 2013) [52] | MARKO-D | Measure not published in English |
|  | Minneapolis Kindergarten Assessment (Betts, et al., 2009) [53] | MKA | Original measure/items not freely available |
|  | Monitoring Basic Skills Progress - Math Computation (2nd edition) probes; Math Concepts and Applications (Fuchs, et al., 1998) [54] | MBSP | Failed to contact authors |
|  | Nonsymbolic numerosity comparison (Patro & Haman, 2012) [55] |  | Not a measure of early numeracy |
|  | Number Knowledge Test (Okamoto & Case, 1996) [56] | NKT | Failed to contact authors |
|  | Number line assessment 0-20; Number line assessment 0-100 (Clarke et al. 2020) [57] | NLA 0–20  NLA 0-100 | No data on content validity |
|  | Number Sense in Kindergarten (Lago & DiPerna, 2010) [58] |  | No data on content validity |
|  | Number Sense Screener (Jordan, et al., 2012) [59] | NSS | No data on content validity |
|  | Numeracy Achievement Instrument (Looveer & Mulligan, 2009) [60] | NAI | Original measure/items not freely available |
|  | Online early numeracy test (Rausch, & Pasztor, 2017) [61] |  | No data on content validity |
|  | Osnabrück test of number concept development (Van Luit, et al., 2001) [62] | OTZ | Failed to contact authors |
|  | Otis-Lennon school ability test 8 (Otis, 1988) [63] | OLSAT8 | Original measure/items not freely available |
|  | Patterning (Rittle-Johnson, et al., 2013) [64] | Patterning | No factor analysis or Rasch analysis |
|  | Performance Indicators in Primary Schools (Godfrey & Galloway, 2004) [65] | PIPS | Original measure/items not freely available |
|  | Pictorial Test of Intelligence, 2^nd^ edition (Eaves & Williams, 2005) [66] | PTI-2 | Original measure/items not freely available |
|  | Preschool mathematics: five experimental (Polignano & Hojnoski, 2012) [67] | CBM | No factor analysis or Rasch analysis |
|  | Preschool Numeracy Indicators (Floyd, et al., 2006) [68] | PNIs | Original measure/items not freely available |
|  | The Primary Mathematics Assessment (Brendefur, et al., 2015) [69] | PMA | No data on content validity |
|  | The Primary Mathematics Assessment-Diagnostic; The Primary Mathematics Assessment-Screener, K-2 (Brendefur, et al., 2018) [70] | PMA-D PMA-S | Original measure/items not freely available |
|  | Process Assessment of the Learner, 2^nd^ edition (Berninger, 2007) [71] | PAL-II | Original measure/items not freely available |
|  | Progress monitoring measures (Salaschek & Souvignier, 2013) [72] |  | No factor analysis or Rasch analysis |
|  | The research-based early maths assessment – short form (Weiland, et al., 2012) [73] | REMA-short | Original measure/items not freely available |
|  | ROOTS Assessment of Early Number Skills (Doabler, et al., unpublished) [74] | RAENS | Failed to contact authors |
|  | Speeded-performance task (Janssen, et al., 1999) [75] |  | Not a measure of early numeracy |
|  | Stanford Achievement Teset Series, 10^th^ edition (Stanford Achievement Test Series, 2002) [76] | SAT10 | Original measure/items not freely available |
|  | Teaching Rating Scale Early Numeracy (Vessonen, et al., 2023) [77] | TRS-EN | Measure not published in English |
|  | Test of Early Mathematics Ability (Ginsburg & Baroody, 2003) [78] | TEMA-3 | No data on content validity |
|  | Test of Early Number and Arithmetic (Bojorque, et al., 2015) [79] | TENA | No Factor analysis or Rasch analysis |
|  | Test of Early Numeracy (Clarke & Shinn, 2002) [80] | TEN | No factor analysis or Rasch analysis |
|  | Utrecht Early Mathematical Competence Test (Barbas, et al., 2008) [81] | ENT | Manual not published in English |
|  | Utrecht Early Numeracy Test (Charitaki, et al., 2023) [82] | ENT | Measure not published in English |
|  | Wechsler Individual Achievement Test, 4^th^ edition (Breaux, 2020) [83] | WIAT-4 | Original measure/items not freely available |
|  | Wide Range Achievement Test 5^th^ edition (Wilkinson & Robertson, 2017) [84] | WRAT-5 | Original measure/items not freely available |
|  | Wide Range Achievement Test 5^th^ edition, expanded (Robertson, 2001) [85] | WRAT-5 expanded | Original measure/items not freely available |

References

1. Messer, M.A., *Academic Achievement Battery: Comprehensive Form*. 2014: PAR.

2. Wheeler, C.E., et al., *Assessment Manual.* 2019.

3. Pearson, N., *AIMSwebplus technical manual.* Bloomington, MN: Author, 2017.

4. ten Braak, D. and I. Størksen, *Psychometric properties of the Ani Banani Math test.* European Journal of Developmental Psychology, 2021. **18**(4): p. 610-628.

5. Gilmore, C.K., S.E. McCarthy, and E.S. Spelke, *Symbolic arithmetic knowledge without instruction.* Nature, 2007. **447**(7144): p. 589-591.

6. Harris, F., et al., *Validation of a two-factor model of the Best Start Kindergarten Assessment of literacy and numeracy.* The Australian journal of education, 2018. **62**(1): p. 36-48.

7. Dumont, R. and J.O. Willis, *Boehm Test of Basic Concepts–Third Edition.* Encyclopedia of Special Education, 2008: p. 314-315.

8. Boehm, A.E., *Boehm Test of Basic Concepts, Preschool: Boehm 3*. 2000: Psychological Corporation.

9. Bracken, B.A., *Bracken Basic Concept Scale--: Receptive.* 2006.

10. Jordan, N.C., J. Glutting, and C. Ramineni, *The importance of number sense to mathematics achievement in first and third grades.* Learning and individual differences, 2010. **20**(2): p. 82-88.

11. Milburn, T.F., et al., *Dimensionality of preschoolers’ informal mathematical abilities.* Early childhood research quarterly, 2019. **47**: p. 487-495.

12. Clements, D.H., J.H. Sarama, and X.H. Liu, *Development of a measure of early mathematics achievement using the Rasch model: the Research‐Based Early Maths Assessment.* Educational Psychology, 2008. **28**(4): p. 457-482.

13. Csapó, B., G. Molnár, and J. Nagy, *Computer-based assessment of school readiness and early reasoning.* Journal of educational psychology, 2014. **106**(3): p. 639.

14. Greisen, M., et al., *Taking language out of the equation: The assessment of basic math competence without language.* Frontiers in psychology, 2018. **9**: p. 1076.

15. Seethaler, P.M. and L.S. Fuchs, *Using curriculum-based measurement to monitor kindergarteners’ mathematics development.* Assessment for Effective Intervention, 2011. **36**(4): p. 219-229.

16. Vanderheyden, A.M., et al., *Development and validation of curriculum-based measures of math performance for preschool children.* Journal of Early Intervention, 2004. **27**(1): p. 27-41.

17. VanDerHeyden, A.M., et al., *The reliability and validity of curriculum-based measurement readiness probes for kindergarten students.* School Psychology Review, 2001. **30**(3): p. 363-382.

18. Hammill, D.D., R.L. McGhee, and D.J. Ehrler, *Detroit tests of learning abilities*. 2018: Pro-ed, an International Publisher.

19. Krajewski, K., P. Küspert, and W. Schneider, *Deutscher Mathematiktest für erste Klassen: DEMAT 1*. 2002: Beltz Test.

20. Lesiak, W.J. and J. Lesiak, *Developmental Tasks for Kindergarten Readiness: An Assessment of Abilities and Skills in Preschool Children to Determine Kindergarten Readiness*. 1978: Clinical Psychology Publishing Company.

21. Newcomer, P., *Diagnostic Achievement Battery, 4th edition*. 2014, Austin, Texas: Pro-Ed.

22. Alam, S. and A. Dubé, *Measuring Digital Home Numeracy Practice: A Scale Development and Validation Study.* Journal of Research in Childhood Education, 2023. **37**(2): p. 310-340.

23. Baek, S.-G. and K.J. Kim, *The effect of dynamic assessment based instruction on children’s learning.* Asia Pacific Education Review, 2003. **4**(2): p. 189-198.

24. DiPerna, J., P. Morgan, and P. Lei, *Development of Early Arithmetic, Reading, and Learning Indicators for Head Start (The EARLI Project).* Semi-Annual Performance Report to the US Department of Health and Human Services Administration for Children and Families (Award No. 90YF0047/01), 2007.

25. Lei, P.-W., et al., *Developing short forms of the EARLI numeracy measures: Comparison of item selection methods.* Educational and Psychological Measurement, 2009. **69**(5): p. 825-842.

26. Clarke, B. and M.R. Shinn, *A preliminary investigation into the identification and development of early mathematics curriculum-based measurement.* School Psychology Review, 2004. **33**(2): p. 234-248.

27. Lee, Y.-S., et al., *Item-level and construct evaluation of early numeracy curriculum-based measures.* Assessment for Effective Intervention, 2012. **37**(2): p. 107-117.

28. Lembke, E.S., et al., *Establishing technically adequate measures of progress in early mathematics*. 2008, University of Minnesota, College of Education and Human Development ….

29. Thomas, A., et al., *Early Numeracy Assessment In French preschool: structural analysis and links with children's characteristics.* International Journal of Early Years Education, 2023. **31**(4): p. 1018-1035.

30. Chard, D.J., et al., *Using measures of number sense to screen for difficulties in mathematics: Preliminary findings.* Assessment for Effective Intervention, 2005. **30**(2): p. 3-14.

31. Lembke, E. and A. Foegen, *Identifying early numeracy indicators for kindergarten and first‐grade students.* Learning Disabilities Research & Practice, 2009. **24**(1): p. 12-20.

32. Conoyer, S.J., A. Foegen, and E.S. Lembke, *Early numeracy indicators: Examining predictive utility across years and states.* Remedial and Special Education, 2016. **37**(3): p. 159-171.

33. Lopez-Pedersen, A., *On the Trail of Early Numeracy Skills: Understanding, identifying and ameliorating young children’s early numeracy skills. A multimethod approach.* 2020.

34. Methe, S.A., J.C. Begeny, and L.L. Leary, *Development of conceptually focused early numeracy skill indicators.* Assessment for Effective Intervention, 2011. **36**(4): p. 230-242.

35. Aunio, P., et al., *The early numeracy test in finnish: Children's norms.* Scandinavian Journal of Psychology, 2006. **47**(5): p. 369-378.

36. Torbeyns, J., et al., *Development of early numeracy in 5-to 7-year-old children: A comparison between Flanders and the Netherlands.* Educational Research and Evaluation, 2002. **8**(3): p. 249-275.

37. David, C., A. Dobrean, and J. HANS VAN LUIT, *Psychometric properties of Early Numeracy Test in Romanian language Preliminary data.* Transylvanian Journal of Psychology, 2015. **16**(1).

38. Feifer, S. and H.K. Clark, *Feifer assessment of Mathematics (FAR)*. 2016, PAR.

39. Williams, K., *GMADE Group mathematics assessment and diagnostic evaluation. Assessment and diagnostic evaluation. Technical manual*. 2004, Circe Pines, MN: AGS Publishing.

40. Jiménez, J. and S. de León, *Indicadores de progreso de aprendizaje en matemáticas (IPAM)-2º curso de educación primaria [Indicators of basic early math skills (IPAM)-2nd grade of primary school].* Modelo de respuesta a la intervención. Un enfoque preventivo para el abordaje de las dificultades específicas de aprendizaje, 2019.

41. de León, S.C., et al., *Identification of Spanish third graders at risk of math problems: Usefulness of number sense based screening measures.* Psychology in the Schools, 2021. **58**(7): p. 1416-1431.

42. Hojnoski, R. and R. Floyd, *Individual growth and development indicators of early numeracy (IGDIs-EN). Minneapolis, MN: Early Learning Labs*. 2013.

43. Kaufman, A.S., *Kaufman Survey of Early Academic and Language Skills: K-SEALS*. 1993: American Guidance Service.

44. Frame, L.B., S.M. Vidrine, and R. Hinojosa, *Kaufman Test of Educational Achievement.* Journal of Psychoeducational Assessment, 2016.

45. Kaufman, A., *Kaufman Test of Educational Achievement, Brief Form (KTEA-3 Brief)*. 2015, Bloomington, MN: Pearson.

46. Connolly, A.J., *KeyMath 3: diagnostic assessment*. 2007: Pearson San Antonio, TX.

47. Gomez, J.A., J.L. Brown, and E. Spier, *Factor structure, measurement invariance, and construct validity of MELQO measure of child development and early learning in Laos.* International Journal of School & Educational Psychology, 2022. **10**(2): p. 181-207.

48. Arendasy, M., M. Sommer, and I. Ponocny, *Psychometric approaches help resolve competing cognitive models: When less is more than it seems.* Cognition and Instruction, 2005. **23**(4): p. 503-521.

49. Erford, B.T., et al., *Reliability and validity of the Math Essential Skill Screener—Elementary Version (MESS‐E).* Psychology in the Schools, 1998. **35**(2): p. 127-135.

50. Lee, Y.-S. and E. Lembke, *Developing and evaluating a kindergarten to third grade CBM mathematics assessment.* ZDM, 2016. **48**(7): p. 1019-1030.

51. Reynolds, C., J. Voress, and R. Kamphaus, *Mathematics fluency and calculation tests examiners manual.* Austin, TX: Pro-Ed, 2015.

52. Ricken, G., L. Balzer, and A. Fritz, *Mathematik-und Rechenkonzepte im Vorschulalter-Diagnose: MARKO-D*. 2013: Hogrefe Göttingen.

53. Betts, J., M. Pickart, and D. Heistad, *Construct and Predictive Validity Evidence for Curriculum-Based Measures of Early Literacy and Numeracy Skills in Kindergarten.* Journal of psychoeducational assessment, 2009. **27**(2): p. 83-95.

54. Fuchs, L., C. Hamlett, and D. Fuchs, *Monitoring basic skills progress manual*. 1998, Austin, TX: PROED.

55. Patro, K. and M. Haman, *The spatial–numerical congruity effect in preschoolers.* Journal of experimental child psychology, 2012. **111**(3): p. 534-542.

56. Okamoto, Y. and R. Case, *Number knowledge test.* Monographs of the Society for Research in Child Development, 1996. **61**(1-2): p. 27-58.

57. Clarke, B., et al., *Exploring the promise of a number line assessment to help identify students at-risk in mathematics.* Assessment for effective intervention, 2020. **45**(2): p. 151-160.

58. Lago, R.M. and J.C. DiPerna, *Number sense in kindergarten: A factor-analytic study of the construct.* School Psychology Review, 2010. **39**(2): p. 164-180.

59. Jordan, N.C., J.J. Glutting, and N. Dyson, *Number sense screener™(NSS™) User's guide, k–1, research edition*. 2012, Baltimore: Brookes Publishing.

60. Looveer, J. and J. Mulligan, *The efficacy of link items in the construction of a numeracy achievement scale—from kindergarten to year 6.* J Appl Meas, 2009. **10**: p. 247-265.

61. Rausch, A. and A. Pásztor, *Exploring the possibilities of online assessment of early numeracy in kindergarten.* 2017.

62. van Luit, J.E., B.A. van de Rijt, and K. Hasemann, *Osnabrücker Test zur Zahlbegriffsentwicklung: OTZ*. 2001: Hogrefe, Verlag für Psychologie.

63. Otis, A.S., *Otis-Lennon school ability test: OLSAT*. 1988: Psychological Corporation.

64. Rittle-Johnson, B., et al., *Emerging understanding of patterning in 4-year-olds.* Journal of Cognition and Development, 2013. **14**(3): p. 376-396.

65. Godfrey, J.R. and A. Galloway, *Assessing early literacy and numeracy skills among Indigenous children with the Performance Indicators in Primary Schools test.* Issues in Educational Research, 2004. **14**(2): p. 144-155.

66. Eaves, R.C. and T.O. Williams, *Pictorial Test of Intelligence-Second Edition.* Assessment for Effective Intervention, 2005. **30**(3): p. 77-84.

67. Polignano, J.C. and R.L. Hojnoski, *Preliminary Evidence of the Technical Adequacy of Additional Curriculum-Based Measures for Preschool Mathematics.* Assessment for effective intervention, 2012. **37**(2): p. 70-83.

68. Floyd, R.G., R. Hojnoski, and J. Key, *Preliminary evidence of the technical adequacy of the preschool numeracy indicators.* School Psychology Review, 2006. **35**(4): p. 627-644.

69. Brendefur, J., et al., *Developing a comprehensive mathematical assessment tool to improve mathematics intervention for at-risk students.* International Journal for Research in Learning Disabilities, 2015. **2**(2): p. 65-90.

70. Brendefur, J.L., et al., *Developing a multi-dimensional early elementary mathematics screener and diagnostic tool: the primary mathematics assessment.* Early childhood education journal, 2018. **46**(2): p. 153-157.

71. Berninger, V., *Process Assessment of the Learner Second Edition: Diagnostics for Reading and Writing (PAL-II)*. 2007, San Antonio, TX: NCS, Pearson.

72. Salaschek, M. and E. Souvignier, *Web-Based Progress Monitoring in First Grade Mathematics.* Frontline Learning Research, 2013. **1**(2): p. 53-69.

73. Weiland, C., et al., *Early mathematics assessment: Validation of the short form of a prekindergarten and kindergarten mathematics measure.* Educational Psychology, 2012. **32**(3): p. 311-333.

74. Doabler, C., B. Clarke, and H. Fien, *Roots assessment of early numeracy skills (raens).* Unpublished measurement instrument. Center on Teaching and Learning, University of Oregon. Eugene, OR, 2012.

75. Janssen, R., et al., *Simple mental addition in children with and without mild mental retardation.* Journal of Experimental Child Psychology, 1999. **74**(3): p. 261-281.

76. Test, S.A., *Stanford Achievement Test Series*. 2002, San Antonio, TX: Pearson Assessment.

77. Vessonen, T., et al., *Validating the early numeracy teacher rating scale for preschoolers (TRS-EN).* European Early Childhood Education Research Journal, 2023. **31**(2): p. 205-224.

78. Ginsburg, H. and A. Baroody, *Test of Early Mathematics Ability, 3rd Edn (TEMA-3).* Austin, TX: Pro-Ed, 2003.

79. Bojorque, G., et al., *Early number and arithmetic performance of Ecuadorian 4-5-year-olds.* Educational Studies, 2015. **41**(5): p. 565-586.

80. Clarke, B. and M.R. Shinn, *Test of early numeracy (TEN): Administration and scoring of AIMSweb early numeracy measures for use with AIMSweb.* Eden Prairie, MN: Edformation Inc, 2002.

81. Barbas, G., et al., *Utrecht early mathematical competence test.* Thessaloniki: Greek Ministry of Education, 2008.

82. Charitaki, G., S. Soulis, and A. A, *Factor structure of early numeracy: evaluation of a measurement model in greek-speaking children with intellectual disabilities.* International Journal of Developmental Disabilities, 2023. **69**(4): p. 505-514.

83. Breaux, K.C., *WIAT 4: Wechsler Individual Achievement Test Technical & Interpretive Manual Fourth Edition*. 2020: NCS Pearson.

84. Wilkinson, G. and G. Robertson, *Wide range achievement test—Fifth edition (WRAT-5).* San Antonio, TX: Psychological Corporation, 2017.

85. Robertson, G.J., *Wide range achievement test-expanded edition.* Wilmington, DE: Wide Range, 2001.
