# Supplementary material for: Content validity of measures in early numeracy in children up to eight years: A COSMIN systematic review
Source: PLoS One. 2024 Sep 19;19(9):e0308874. doi: 10.1371/journal.pone.0308874 (PMC11412641; doi:10.1371/journal.pone.0308874)
Supplement: S4 Table — Description of the development and content validity studies on included measures. (DOCX) [file pone.0308874.s004.docx]

**S4 Table. Study details.** Description of the development and content validity studies on included measures.

| **Instrument**  *(acronym)* | **Source**  *(type)* | **Purpose of Study** | **Study Population** | **Age:** *range [R] +/or Mean [M] +/or Standard Deviation [SD]* | |
| --- | --- | --- | --- | --- | --- |
| **CIRCLE Progress Monitoring-Math Subtest**  (CPM) | Assel et al. 2020 (study) (1) | (1) What is the dimensionality of the new subtest? (2) To what extent does the subtest demonstrate internal consistency and test-retest reliability? (3) To what extent does the subtest demonstrate concurrent, predictive, and divergent validity in terms of relations with other standardised, norm-referenced math measures? | N = 383 (Stage – initial validation sample): (I) research assistants.  N = 3961 (Stage – large validation sample): (II) teachers. | Initial validation sample, M (SD): 4.9 (0.4) years  Large validation sample, M (SD): 4.40 (0.45) years. | |
| **Core Early Grade Mathematics Assessment**  (Core EGMA) | Platas et al. 2016 (study) (2);  Platas et al. 2014 (Toolkit) (2);  RTI International 2009 (Framework) (3) | Describes the development and intended use of the Early Grades Mathematics Assessment (EGMA). Provide examples of where EGMA results have been used to evaluate the effectiveness of educational policies, curricular reforms or programs, and instructional practices and interventions. | N = 7923_Ghana_  N = 100_Suriname_  N = NR_Kenya_  (Information relating to stage or instrument administrators NR). | Ghana: age NR, grade 2.  Suriname: age NR, grades 3–4.  Kenya; age NR, grades 1–2 | |
| **Early Learning Outcomes Measure**  (ELOM) | Dawes et al. 2016 (study) (4) | “…to construct a sample that was likely to be as representative as possible of children eligible to enter Grade R in January 2016, drawn from across South Africa’s socio-economic distribution, and including five major language groups” (Dawes et al., 2016, p.12). | N = 1476 (Stage – age validation study): (I) field managers and senior assessors. | R: 50–69 months | |
| **International Development and Early Learning Assessment**  (IDELA) | Pisani et al. 2018 (study) (5);  Pisani et al. 2015 (technical working paper) (6) | “…to investigate different aspects of item functioning and arrive at the final set of items” (Pisani et al., 2018, p.4).  “…to develop a holistic, rigorous, open source instrument that is feasible and easily adapted to different national and cultural contexts” (Pisani et al., 2015, p.4). | N_Bangladesh_ = 594. N_Bhutan_ = 99. N_Egypt_ = 444. N_Ethiopia_= 682. N_Ethiopia_ = 100. N_Indonesia_ = 148. N_Malawi_ = 748. N_Mali_ = 1260. N_Mozambique_ = 161. N_Pakistan_ = 473.N_Rwanda_ = 722 N_Zambia_ = 318. (Stage – pilot, adaptation & selection of final items): (I) various stakeholders including community members, educators and researchers (Pisani et al., 2018).  N = 5304 (total of studies listed above; Pisani et al., 2015). | R: 3–6 years | |
| **Preschool Early Numeracy Skills Screener-Brief version**  (PENS-B) | Purpura et al. 2015 (study) (7);  Purpura & Lonigan 2015 (study) (8);  Purpura, 2021 (manual) (9) | To design and evaluate a brief early numeracy skills screening tool. | N = 393 (Stage – measure development): (I) people with, or in the process of completing, bachelor degrees who completed 2-3 hour training sessions.  N = 129 (Stage - measure validation): (I) psychology graduates with, or in the process of completing, doctoral degrees who completed 1–2-hour training sessions and were required to demonstrate proficiency. | R: 3.63–5.85 years  M (SD): 4.75 (0.75) years  R: 3.13–5.98 years  M (SD): 4.79 (0.49) years | |
| **Tools for Early Assessment in Math, Danish version**  (DK-TEAM) | Sjoe et al. 2019 (study) (10) | To develop a short version of the mathematics test TEAM that could be administered by childcare workers to Danish children aged 3–6years. | N = 179 (Stage – pilot study): (I) child-care workers, neurospsychologists, test developer, linguist, graphical designer, TEAM developers.  N = 5584 (Stage – items used in representative sample): (II) child-care workers. | R: 3–6 years |  |

*Notes*. CV = convergent validity; IC = internal consistency; IRR = inter-rater reliability; T-RR = test-retest reliability.

**References**

1. Assel MA, Montroy JJ, Williams JM, Foster M, Landry SH, Zucker T, et al. Initial Validation of a Math Progress Monitoring Measure for Prekindergarten Students. Journal of Psychoeducational Assessment. 2020;38(8):1014-32.

2. Platas LM, Ketterlin-Geller LR, Sitabkhan Y. Using an assessment of early mathematical knowledge and skills to inform policy and practice: Examples from the early grade mathematics assessment. International Journal of Education in Mathematics, Science and Technology. 2016;4(3):163-73.

3. EdData I. Early Grade Mathematics Assessment (EGMA): A Conceptual Framework Based on Mathematics Skills Development in Children. 2009.

4. Dawes A, Biersteker L, Girdwood E, Snelling M, Tredoux C, Anderson K, et al. Early learning outcomes measure. Technical manual. Cape Town: The Innovation Edge; 2016.

5. Pisani L, Borisova I, Dowd AJ. Developing and validating the international development and early learning assessment (IDELA). International Journal of Educational Research. 2018;91:1-15.

6. Pisani L, Borisova I, Dowd AJ. International development and early learning assessment technical working paper. Save the Children; 2015.

7. Purpura DJ, Reid EE, Eiland MD, Baroody AJ. Using a brief preschool early numeracy skills screener to identify young children with mathematics difficulties. School Psychology Review. 2015;44(1):41-59.

8. Purpura DJ, Lonigan CJ. Early numeracy assessment: The development of the preschool early numeracy scales. Early education and development. 2015;26(2):286-313.

9. Purpura DJ. PENS: Preschool Numeracy Screener. Examiners Manual.: Pro-Ed; 2021.

10. Sjoe NM, Bleses D, Dybdal L, Tideman E, Kirkeby H, Sehested KK, et al. Short Danish Version of the Tools for Early Assessment in Math (TEAM) for 3–6-Year-Olds. Early Education and Development. 2019;30(2):238-58.
